# Medical Section of the Royal Society of New South Wales

**Published:** 1888-01

**Authors:** 


### MEDICAL SECTION OF THE ROYAL SOCIETY OF NEW SOUTH WALES.

Meeting held in the Society's Rooms, Sydney, on August 19, 1887.

Members present: Dr. Sydney Jones, President, in the chair, Drs. Knaggs, Skirving, Goode, Ross, Faithfull, Chambers, Chisholm, Cragi, Power, Milford, Hankins, Lyden, Martin, Worrall, Brady, Twynam, Clune, Fiaschi, Garrett, MacLaurin, Mander Jones, Ellis, West, Anderson Stuart, and MacCormick and Jenkins (Hon. Secretaries.)

Dr. Knaggs exhibited for Dr. Roth a new spirometer and dynamometer, and explained the mechanism of both.

Dr. Sydney Jones regarded these instruments of little practical value in individual cases, but useful when statistics of large numbers of cases were kept, as tending to show the physique of a nation.

Drs. Milford, Garrett, Faithfull, Crago and Skirving, remarked that neither instrument was made use of in the insurance societies of which they were the medical officers.

Dr. Chambers then exhibited specimens:

(1) Hair-pin from urethra of girl aged 13. The hair-pin was inserted by the girl, and voided without surgical interference.

(2) New growth from "vulva," removed by Paquelin cautery. Of doubtful nature, but probably epithelioma or condyloma.

(3) *Uterine Fibroid*, from a woman aged 42. The whole uterus and appendages were removed, and the patient made a perfect recovery. A small intra-uterine polypus protruded through cervix. The difficulties in diagnosis were many and great before the operation.

Dr. Brady gave history of a child with stone in bladder formed round a pin. The child was only three years old.

Dr. Garrett exhibited the middle finger of his left hand, and the forefinger of his right, both in a condition of dry gangrene from accidental application of pure carbolic acid.

Dr. Goode related a similar case from a 1 in 20 solution of carbolic lotion, and Dr. MacCormick made some remarks on the anæsthetic and caustic action of carbolic acid, and the necessity of using warm water for diluting it.

Meeting held on September 16, Dr. Sydney Jones in the chair. There were also present: Drs. Twynam, Chisholm, Goode, Waugh, Mander Jones, Faithfull, MacCulloch, Quaife, MacCormick, Jenkins, Sir Alfred Roberts, Brady, Worrall, Garrett, Skirving, Fiaschi and Caruthers.

The minutes of previous meeting having been read and confirmed,

Dr. Twynam read papers on two cases of amputation at the hip joint, and both patients were exhibited. One excited great interest, as the diseased bone which necessitated the operation was caused by caisson fever, contracted March 6, 1882, the patient working under a pressure of 45 pounds to the square inch.

Sir Alfred Roberts made some remarks, having seen the patient in the interest of the Government some time back, and that then there was exquisite tenderness over the right thigh, muscular pains, lung troubles and great deviations in the temperature.

Dr. Jones also joined in the discussion, and congratulated Mr. Twynam on the successful issue of his cases.

Dr. MacCulloch exhibited a patient with a marked curvature of left tibia. There had been a previous fracture of the fibula, but no fracture of the tibia. The bending commenced some considerable time after the accident. Drs. Brady, Waugh, and MacCormick joined in the discussion.

Dr. MacCormick exhibited a small piece of bone tipped with cartilage that he had removed from the knee joint.

Dr. Sydney Jones exhibited a peculiar growth he had removed from the anterior bone of axilla," an epithelial growth, with colloid degeneration."

Drs. Twynam and Skirving joined in the discussion.

---

Meeting held on October 21, 1887. Present: Dr. Sydney Jones in the chair, Drs, Blaxland, MacAllister, Faithfull, Kendall, Ross, Roth, Anderson Stuart, Garrett, Crago, Lyden, Waugh, Theophilus Jones, Chisholm, Goode, Deck, Knaggs, Hankins, Twynam, Worrall, Brady, Power, Wright, Phillips, MacCulloch, Fiaschi, Quaife, MacCormick, and Jenkins.

Dr. Chisholm read notes on a case of gastrotomy, and exhibited the patient, who fed himself through a glass funnel with milk. The true cause of the œsophageal stricture was obscure.

Dr. Hankins gave history of three cases of gastrotomy, two in adults, both of whom died, one from symptoms of peritonitis, and the other from inanition; and the third, a child. The smallest bougie could not be passed. The first stage of the operation was performed, and then a No. 1 catheter was passed, and it was found unnecessary to perform the second stage.

Drs. MacCormick, Brady, Anderson Stuart and Sydney Jones, joined in the discussion.

Dr. Waugh then exhibited a case of vicious union of the tibia—the lower half of tibia and fibula set at right angles to upper half. He was treated in the Melbourne Hospital, but left three weeks after admission, and was warned of the dangers.

Dr. Goode read a paper on a case of "Excision of the Rectum," and showed the case. Drs. Twynam and Sydney Jones made remarks as to the benefits of the operation, the methods, the prevention of bleeding, etc.

Dr. Sydney Jones then read, for Dr. Mander Jones, a paper on a case of "Intestinal Obstruction" in a woman aged 22, following inflammation after abortion. Dr. Sydney Jones opened the abdomen, and found the obstruction due to a dense band connecting the ileum and colon, with a mesenteric gland involved. The adhesion was cut through, and the patient made an excellent recovery. Drs. Ross, Crago, MacCormick, Twynam, and Worrall joined in discussion, the latter urging immediate operative interference in cases with urgent symptoms.

Dr. Sydney Jones replied.

Dr. Goode exhibited—

(1) Strangulated femoral hernia.

(2) An enormous fatty tumor.

